# EEG Changes in Time and Time-Frequency Domain During Movement Preparation and Execution in Stroke Patients

**DOI:** 10.3389/fnins.2020.00827

**Published:** 2020-08-20

**Authors:** Hai Li, Gan Huang, Qiang Lin, Jiangli Zhao, Qiang Fu, Le Li, Yurong Mao, Xijun Wei, Wanzhang Yang, Bingshui Wang, Zhiguo Zhang, Dongfeng Huang

**Affiliations:** ^1^Neurorehabilitation Laboratory, Department of Rehabilitation Medicine, Shenzhen Hospital, Southern Medical University, Shenzhen, China; ^2^Department of Rehabilitation Medicine, Guangdong Engineering Technology Research Center for Rehabilitation Medicine and Clinical Translation, The First Affiliated Hospital, Sun Yat-sen University, Guangzhou, China; ^3^Guangdong Provincial Key Laboratory of Biomedical Measurements and Ultrasound Imaging, School of Biomedical Engineering, Health Science Center, Shenzhen University, Shenzhen, China; ^4^Department of Rehabilitation Medicine, The Fifth Affiliated Hospital of Guangzhou Medical University, Guangzhou, China; ^5^Ira A. Fulton Schools of Engineering, Arizona State University, Tempe, AZ, United States; ^6^Department of Rehabilitation Medicine, The Seventh Affiliated Hospital, Sun Yat-sen University, Guangzhou, China

**Keywords:** stroke, electroencephalogram, movement-related cortical potential, event-related desynchronization, movement preparation, movement execution

## Abstract

This study investigated electroencephalogram (EEG) changes during movement preparation and execution in stroke patients. EEG-based event-related potential (ERP) technology was used to measure brain activity changes. Seventeen stroke patients participated in this study and completed ERP tests that were designed to measure EEG changes during unilateral upper limb movements in preparation and execution stages, with Instruction Response Movement (IRM) and Cued Instruction Response Movement (CIRM) paradigms. EEG data were analyzed using motor potential (MP) in the time domain and the mu-rhythm and beta frequency band response mean value (R-means) in the time–frequency domain. In IRM, the MP amplitude at Cz was higher during hemiplegic arm movement than during unaffected arm movement. MP latency was shorter at Cz and the contralesional motor cortex during hemiplegic arm movement in CIRM compared to IRM. No significant differences were found in R-means among locations, between movement sides in both ERP tests. This study presents the brain activity changes in the time and time–frequency domains in stroke patients during movement preparation and execution and supports the contralesional compensation and adjacent-region compensation mechanism of post-stroke brain reconstruction. These findings may contribute to future rehabilitation research about neuroplasticity and technology development such as the brain–computer interface.

## Introduction

Motor control dysfunction is one of the most common disabilities that result from a stroke (Winstein et al., 2016). Understanding the brain activity changes in the motor cortex during movement preparation and execution is critical for the study of brain plasticity and develop rehabilitation strategies for stroke patients. Animal experiments suggested that after stroke, the central nervous system generates recovery through reconstruction mechanisms such as contralesional hemisphere compensation (Liu et al., 2019), activation of the brain structures surrounding damage, and redistribution of the cortical representative regions (Hara, 2015). The motor cortex was reported to show laterality changes after stroke. During unilateral extremity movements, brain activation was observed to be the strongest in the contralesional motor cortical area in the early stage of a stroke. When the movement pattern gradually normalized through recovery, the brain activation center gradually shifted back to the lesioned side (Liu et al., 2019). Contralesional compensation of the motor cortex showed a positive correlation with the severity of brain damage (Dijkhuizen et al., 2001, 2003; Hsu and Jones, 2006). Another cortical reorganization pattern in stroke rehabilitation, which is adjacent undamaged area compensation, was observed by Starkey and colleagues using antegrade and retrograde tracing techniques and intracortical microstimulation techniques in stroke model rats (Starkey et al., 2012).

Movement-related cortical potentials (MR) are commonly used event-related potential (ERP) measures in the electroencephalogram (EEG) time domain (Peters et al., 2018). The negativity amplitude of MRCPs may reflect energy consumption during movements (Wright et al., 2011) and may show a larger MP amplitude in stroke patients during movement, suggesting an increased energy demand for the injured brain hemisphere (Wiese et al., 2005; Yilmaz et al., 2013). Event-related desynchronization (ERD) reflects the brain’s activity changes that are related to events or movements in the time–frequency domain (Schomer and Lopes da Silva, 2011). ERD occurs not only during movement preparation and execution but also during movement observation, within different frequency band (alpha, beta, and theta, etc.) (Miller et al., 2007; Babiloni et al., 2016, 2017; Angelini et al., 2018). ERD magnitude in the damaged hemisphere in stroke patients showed a positive correlation with the remaining motor ability in the paretic limb (Bartur et al., 2019). In patients with unilateral hemiplegia, damages in the motor area of one hemisphere generally affect the movement function of the contralesion limbs. However, studies on brain injury patients with hemiplegia have found that unilateral brain injury could affect not only the contralateral limb but also the ipsilateral limb motor function (Smutok et al., 1989). EEG studies report that patients with chronic stroke with different lesion locations show different EEG lateralization feature during movement (Park et al., 2016). These studies suggested that lateralization features in behavior and movement-related EEG in patients with unilateral hemiplegia need further investigation, and the lesion location should be considered as an important factor linking the clinical functions and the EEG findings. In our previous study, we combined MRCPs and ERD to investigate the movement preparation and execution in 34 healthy participants and found different lateralization features in the time domain and time–frequency domain. Both hemispheres were involved in the unilateral movement execution stage with evidence from MRCPs and ERD record, while mu-ERD was found to have stronger contralateralization features in the movement preparation stage (Li et al., 2018).

The present study investigated EEG changes during movement preparation and execution in stroke patients in both the time and time–frequency domains. Our hypothesis is that brain activity changes during movement in stroke patients might show specific topographical features in the time and time–frequency domains that differ from healthy people, which reflect the brain reconstruction mechanisms that are found in animal studies.

## Materials and Methods

### Participants

Seventeen stroke patients (14 males, 3 females; mean age, 54.1 ± 10.95 years) participated in this study. There were 10 patients with cerebral infarction and 7 patients with cerebral hemorrhage. The inclusion criteria were as follows: (1) First-time onset stroke patient with a diagnosis given by a neurologist and confirmed by CT or MRI (stroke onset time > 1 month); (2) with unilateral hemiplegia; (3) upper limb Brunnstrom stage ≥ II; (4) right-handed before the stroke onset, self-recognized by the patients and confirmed by the Edinburgh Handedness Inventory (Oldfield, 1971); (5) sufficient cognitive function to follow the researchers’ direction and complete the study, with an MMSE score within the normal range according to education level; and (6) stable general condition that could withstand the ERP laboratory environment and complete the ERP tests. The exclusion criteria were as follows: (1) unstable general condition; severe complications such as congestive heart failure, lower extremity deep venous thrombosis, malignant progressive hypertension, respiratory failure, active liver disease, liver, and kidney dysfunction that may not adapt to the ERP laboratory environment and complete the ERP tests; (2) with severe cognitive dysfunction so that the patient cannot understand and perform experimental tasks; (3) with upper limb muscular–skeletal diseases so that the patient cannot perform movements that are required in this study; (4) a previous history of mental illness or taking any antipsychotic drugs; (5) collapse syndrome so that the patient cannot endure staying in the ERP experiment room; and (6) severe lateral neglect syndrome. More demographic, clinical, and neuropsychological characteristics of the patients are shown in Table 1.

**TABLE 1:** Demographic, clinical, and neuropsychological characteristics of the patients.

**Patient number**	**Age**	**Gender**	**Cause of stroke**	**Hemiplegic side**	**Stroke lesion location**	**Time since onset (month)**	**Upper limb Brunnstrom stage**
01	69	Female	Cerebral hemorrhage	left	Right basal ganglia	16	III
02	55	Male	Cerebral infarction	left	Right basal ganglia Pons	4	VI
03	52	Male	Cerebral infarction	right	Left basal ganglia	1	II
04	58	Male	Cerebral infarction	right	Left Pons	2	V
05	59	Male	Cerebral infarction	right	Left basal ganglia	3	V
06	71	Male	Cerebral infarction	right	Left basal ganglia	1	VI
07	48	Male	Cerebral infarction	right	Left corona radiata and basal ganglia	23	V
08	50	Male	Cerebral hemorrhage	left	Right basal ganglia	9	VI
09	43	Male	Cerebral infarction	left	Right corona radiata basal ganglia	3	VI
10	60	Male	Cerebral hemorrhage	left	Right thalamus	18	VI
11	49	Male	Cerebral hemorrhage	left	Right basal ganglia	9	II
12	72	Male	Cerebral infarction	left	Right basal ganglia	7	III
13	57	Male	Cerebral infarction	left	Multiple lacunar infarction and encephalomalacia in the left basal ganglia	1	VI
14	44	Female	Cerebral hemorrhage	left	Right basal ganglia	3	II
15	58	Male	Cerebral infarction	right	Left basal ganglia	1	II
16	30	Female	Cerebral hemorrhage	right	Left basal ganglia	5	III
17	44	Male	Cerebral hemorrhage	right	Left basal ganglia	24	III

The ethics committee at the First Affiliated Hospital of Sun Yat-sen University pre-approved the study protocol before study implementation [(2013)C-068]. All participants were stroke patients who had clear consciousness and who could express their willingness to participate. The study protocol was explained to all stroke patients before formal participation, and they provided written informed consent. Some of the stroke patients with right hemiplegia were unable to sign an informed consent form because of restricted upper limb function. In this case, an immediate family member signed the informed consent form on their behalf. All participants had no ERP study experience before participation in this study. The ERP experiment is a non-invasive examination. The patients were required to wear an electrode cap and perform some specified movements in the laboratory following the instructions during ERP experiments. The movement task in this study was the wrist movement in the sitting position, which kept the patient’s energy consumption low, and it was not likely to cause increased cardiopulmonary load. Hence, we regard the biosecurity concerns in this study as minimal. However, considering that the experiments were performed in an independent laboratory, in order to ensure that no accidents or physical discomforts occur, the researchers observed and recorded the patient’s behavior through the observation window, where any unexpected situation can be checked in time. In addition, between tests, the researcher routinely asked about the patients’ health conditions and opinions. They were encouraged to take a rest if needed. The patients were told that they had the right to quit the study at any stage, and it would not have any impact on their normal medical services.

### Experimental Settings and Data Acquisition

**FIGURE 1:**
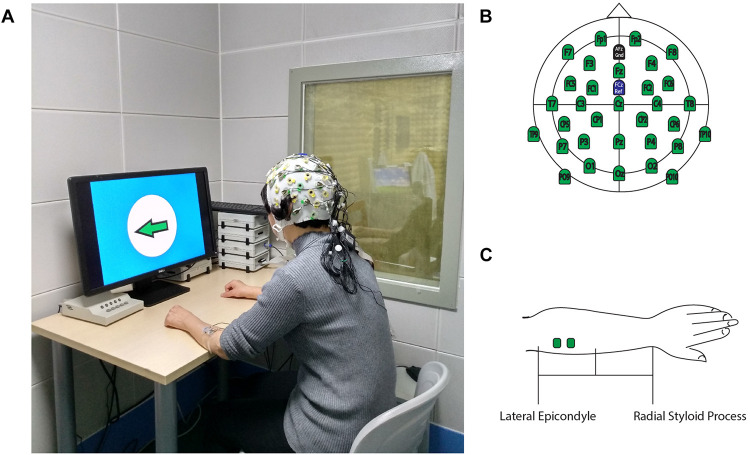
Experimental Setup. **(A)** Participants performed ERP experiments in a shielded room. **(B)** 32-channel EEG electrodes location for the recording cap. **(C)** EMG electrodes location on ECR. This figure adapted from Li et al. (2018).

This study was conducted at the First Affiliated Hospital of Sun Yat-sen University, in the Laboratory of Brain Functional Informatics in the Rehabilitation Medicine Department. The ERP experiment room was insulated against electromagnetic signals and background noise. The shielded room has an observation window, which allows the researcher to observe the behavioral responses of the subject during the ERP experiment, recognize unexpected situations during ERP experiments, and record the number of correct behavioral responses to the experiment instructions. During the experiment, the subject sat in front of a table on which a computer screen was placed, and the screen was about 75 cm from the subject (eye level). The subject’s forearms were placed on the table to maintain a stable sitting position (Figure 1A). The electrode cap that was used to record the EEG signal was a 32-channel actiCap (Herrsching, Germany) that was produced by Brain Products (Munich, Germany). A matching 32-channel BrainAmp Amplifier (Brain Products, Munich, Germany) was used to amplify and record EEG signals. The Ag/AgCl electrode placement was arranged according to the extended international 10–20 system, with references at FCz and ground at AFz (Figure 1B). To measure the wrist extension, a pair of electrodes was placed on each forearm on the extensor carpi radialis (ECR) muscles to record surface EMG signals. Participants received a skin preparation to reduce the impedance between electrodes and the skin. Nuprep Skin Prep Gel (Weaver and Company, Aurora, CO, United States) was used on the skin, and alcohol pads were used to clean off the gel. Then, researchers placed two Ag/AgCl electrodes in the middle of the proximal half of the forearm, which were 2 cm apart (Figure 1C). To ensure data quality, the EEG and EMG electrode impedance was kept under 5 kOhm, and the sampling rate was 1,000 Hz. The input impedance of the EMG single amplifier was 10 MOhm, with a common-mode rejection ratio (CMRR) of > 90 dB and a signal-to-noise ratio of < 1 μVpp. The actual gain range was ± 16.384 mV. A Butterworth filter was used to filter the raw EMG signal, and the low- and high-pass cutoffs were 1,000 and 0.016 Hz, respectively (Merletti, 2018). All EEG and EMG signals were recorded using a BrainVision Recorder (Munich, Germany) synchronously during the ERP experiments, and the results were analyzed offline.

### ERP Paradigms

**FIGURE 2:**
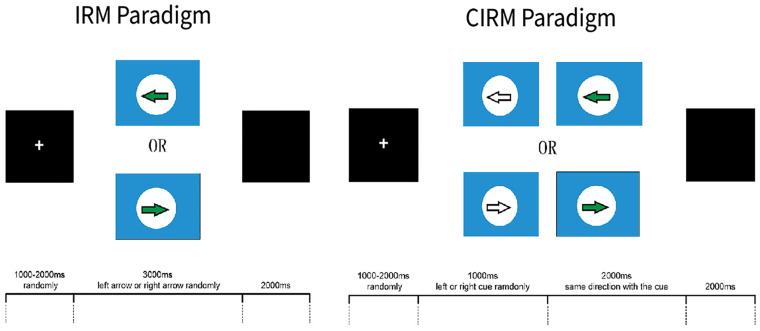
E-Prime ERP experiment paradigms. This figure adapted from Li et al. (2018).

The software E-prime (Psychology Software Tools, Sharpsburg, PA, United States) was used to present the visual instructions and cues in the ERP experiments. Two ERP paradigms were used in this study, which were the Instruction Response Movement (IRM) and Cued Instruction Response Movement (CIRM) (Figure 2). The motor tasks in this study were unilateral wrist extension movements of the left or right forearm, according to the direction that was identified by the visual instructions. Because of impaired motor function, some stroke participants may have difficulty with performing wrist extension. They were required to perform the motor tasks as long as they could or to imagine the movement of the hemiplegic arm if they were not able to perform the movement. The IRM began by presenting a black screen with a white cross (attention point) in the middle (800–1,000 ms, randomly). Then a solid arrow picture (GO signal) was shown on the screen, pointing to the left or the right. The arrow picture lasted for 3,000 ms, and then a black screen was presented that lasted for 2,000 ms. The direction in which the arrow was pointing was random, and it was assigned using the E-prime paradigm, with 40 trials on each side (Jochumsen et al., 2015). Participants were instructed to perform wrist extension with the left or right wrist, which corresponded to the side to which the arrow pointed. Participants were told to perform the movement once for each trial and then rest their arms on the table until the next trial. In the CIRM paradigm, a hollow arrow with the same direction as the GO signal was presented as a visual cue 1 s before the GO signal, and it lasted for 2,000 ms followed by a black screen (2,000 ms). The randomly chosen arrow direction and repetition were the same as for the IRM. The participants were asked to perform wrist extension only after the GO signal. Our previous study found that providing a visual cue containing the movement direction information before the actual GO signal can create a movement preparation status, during which the motor cortex is activated but withholds the motor demand until the movement instruction. This paradigm is particularly suitable to study movement preparation or intention status (Li et al., 2018). The subjects practiced the required movements for 1–2 min before the experiments actually started, and the data were recorded. Between experiments, researchers checked the subjects’ condition, and the subjects could take a short break between trials if necessary. All the subjects completed the ERP experiments in the same order.

### Data Processing

The BrainVision Analyzer 2.1 (Brain Products), Matlab 2016a (The MathWorks Inc., Natick, MA, United States), and Letswave (Version 7^1^) were used to analyze offline EEG data. Letswave is a free software toolbox to analyze the EEG data based on MATLAB language. The EMG add-on component of the BrainVision Analyzer software was used to analyze the EMG data.

The common average was used to re-reference raw EEG data. An independent component analysis (ICA)-based ocular correction was used to remove EEG noise resulting from ocular components. A Butterworth filter (low cutoff, 0.01 Hz; high cutoff, 50 Hz; 12 decimal/octave) was used to filter the noise. Then the EEG data were segmented from −1.2 to 3.0 s relative to the GO signal for both test and baseline-corrected data using −1.2–0 s for the IRM and −1.2– −1.0 s for the CIRM. Trials with the same condition movement were averaged and calculated for MP amplitude and latency. Segmented EEG data were transformed into time–frequency data with averaged continuous wavelet transformation (CWT). Since a frequency-dependent window is used in CWT for the time–frequency estimation, shrinking the interval for baseline correction in the time–frequency domain is suggested (Hu and Zhang, 2019). Baseline correction using data from −1.15 to −0.05 s in the IRM and from −1.15 to −1.05 s in the CIRM was conducted. Time–frequency maps (Mammone et al., 2020) were graphed with the Letswave graphing function. The 8–13-Hz frequency band data were calculated for the mu-rhythm ERD (mu-ERD) (Angelini et al., 2018), and the 13–30-Hz frequency band data were calculated for the beta-rhythm ERD (β-ERD). The phenomenon of ERD/ERS was time locked but non-phase locked. Hence, we chose mean values of the time–frequency response (R-means) for data analysis in the mu and β frequency band in movement execution (0.5–1.5 s) and movement preparation (−0.5–0 s), respectively. The BrainVision Analyzer was used to detect the EMG onset time and the EMG peak data, with onset definition of crossing the threshold at 4 SD from baseline mean in either direction (Yilmaz et al., 2013). The EMG onset time, peak time, and peak amplitude were exported for data analysis.

### Statistical Analysis

EMG onset time, EMG peak time, and EMG peak value were compared by paired sample *t*-test between the lesional and contralesional sides, and between the IRM and CIRM. EEG data at C3, Cz, and C4 were chosen to represent the motor cortex function in the central area and each side (Li et al., 2018). The MP latency and amplitude were analyzed representing the MRCP feature and the mean values of the time–frequency response (R-means) representing time–frequency features. The two-way analysis of variance (ANOVA) was conducted to compare EEG data among locations and sides in each test. The two independent variables were movement side (hemiplegic limb movement vs. unaffected limb movement) and chosen EEG record locations to represent the motor area of each side and the central motor area (lesioned side, Cz, and unaffected side). *Post hoc* tests were conducted to test the differences between locations using Tukey’s method. To test the lateralization feature of EEG components, paired-sample *t*-test was used to test the differences between the lesional and contralesional sides. Paired-sample *t*-test was also used to test the EEG component differences between the IRM and CIRM and the R-means between different frequency bands. The software IBM SPSS statistics 22 (IBM Corporation, Armonk, NY, United States) was used to conduct statistical analyses, and a = 0.05 was set as the statistical significance acceptance level for all tests.

## Results

### EMG

**FIGURE 3:**
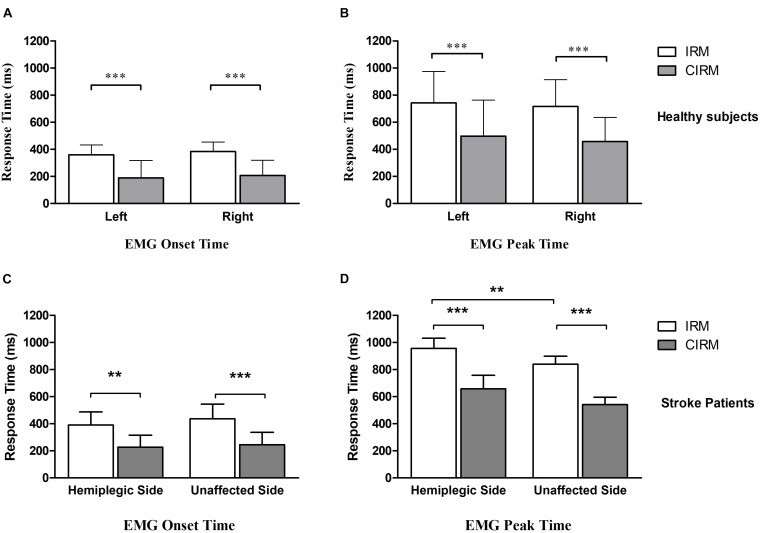
Bar graphs showing average EMG onset time **(A)** and EMG peak time **(B)** without or with cues in IRM and CIRM in healthy subjects and stroke patients. Error bars represent the SD. Significant differences are indicated by asterisks. In healthy subjects, the average EMG onset time and peak time is shorter in CIRM than in IRM and there is no significant difference between sides. In stroke patients: **(C)** The average EMG onset time is shorter in CIRM than in IRM. **(D)** The average EMG peak time is longer on the hemiplegic side than on the unaffected side. The average EMG peak time is shorter in CIRM than in IRM on both sides. ^∗∗^*p* < 0.01, ^∗∗∗^*p* < 0.001.

Because of impaired movement function, six participants did not induce myoelectric activity on the ECR muscle of the paraplegic arm during paraplegic side movement tasks. With those who could perform wrist extension movement and produce EMG signal, the onset time and peak time were shorter for the CIRM than for the IRM on both sides, and the peak time was longer on the hemiplegic side than on the unaffected side for the IRM (*p* < 0.01) (Figure 3). There were no significant differences in the peak value between the two tests or sides.

### MRCPs

**FIGURE 4:**
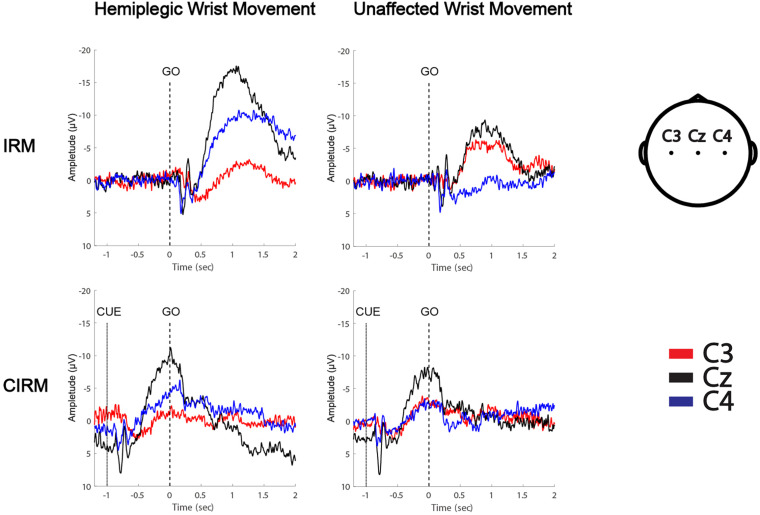
MRCPs of a left hemiplegic stroke patient during wrist movements in IRM and CIRM. Negative upwards.

**FIGURE 5:**
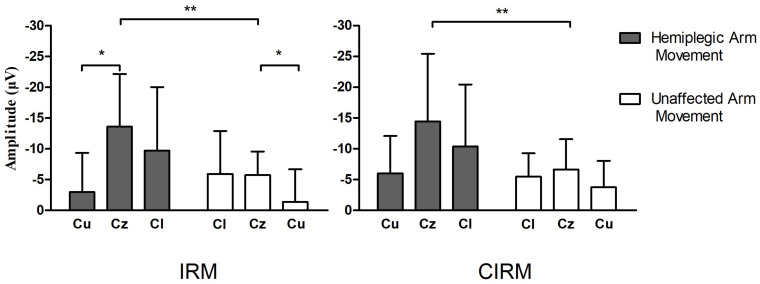
Bar graphs showing average MP amplitude recorded at motor cortex locations during hemiplegic arm movement and unaffected arm movement in IRM and CIRM. Error bars represent the SD. Cl presents the motor cortex on the lesioned side, Cu presents the motor cortex on the unaffected side. Significant differences are indicated by asterisks, *p < 0.05, **p < 0.01. Negative Upwards.

MRCPs during both tests were recorded on all electrodes. C3, Cz, and C4 were chosen for data analysis to investigate the EEG lateralization feature during movements. MP latency and amplitude were calculated from the MRCP waves (Figure 4). The two-way ANOVA was conducted, which examined the effect of the movement side (hemiplegic limb movement vs. unaffected limb movement) and the locations on the EEG components. According to the MRCP data in the IRM, there was no statistically significant interaction between the effects of the movement side and representing the EEG locations on the MP latency [*F*_(2, 96)_ = 1.321, *p* = 0.272]. There was a statistically significant interaction between the effects of the movement side and representing EEG locations on the MP amplitude [*F*_(2, 96)_ = 6.314, *p* = 0.003]. There was a statistically significant difference in the MP amplitude between the hemiplegic limb movement and the unaffected limb movement [*F*_(1, 96)_ = 9.040, *p* = 0.003], and there was a statistically significant difference in the MP amplitude among the EEG locations [F (_2, 96_) = 4.655, *p* = 0.012]. The Tukey *post hoc* test results for the different EEG locations indicated that there is a statistically significant difference between Cz and Cu (unlesioned side) (*p* = 0.013), but not between Cz and Cl (lesioned side) (*p* = 0.063) or between Cl and Cu (*p* = 0.816). According to MRCP data in the CIRM, there was no statistically significant interaction between the effects of the movement side and representing EEG locations on the MP latency [*F*_(2, 96)_ = 0.632, *p* = 0.535]. There was no statistically significant interaction between the effects of movement side and representing EEG locations on the MP amplitude [*F*_(2, 96)_ = 1.892, *p* = 0.158]. There was a statistically significant difference in the MP amplitude between the hemiplegic limb movement and the unaffected limb movement [*F*_(1, 96)_ = 0.9189, *p* = 0.003], and there was no statistically significant difference in the MP amplitude among the EEG locations [*F*_(2, 96)_ = 3.036, *p* = 0.054] (Figure 5). A paired-sample *t*-test between the two paradigms showed that the MP latency was shorter at Cz (*T* = 2.936, *p* = 0.012) and the contralesional motor cortex (*T* = 4.277, *p* = 0.001) during hemiplegic arm movement in the CIRM than in the RIM.

### Time–Frequency Mapping and ERD

**FIGURE 6:**
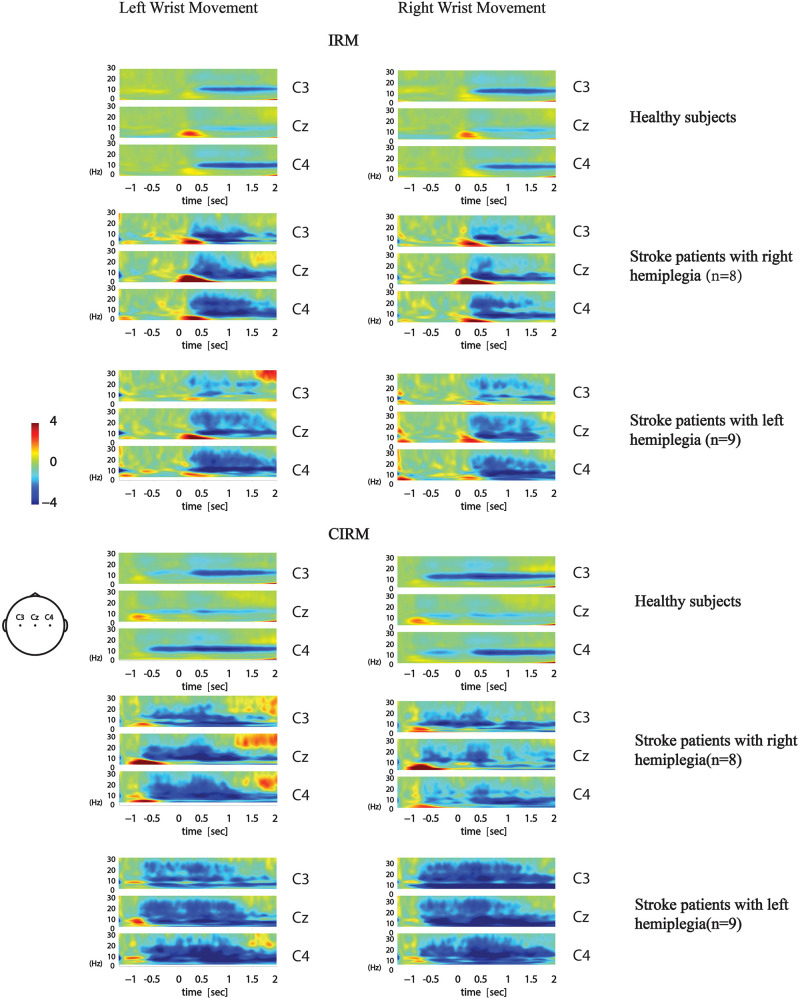
Average time-frequency mapping of stroke patients with hemiplegia during wrist movements in IRM and CIRM. Compared with healthy subjects performing the same ERP paradigms.

The time–frequency EEG data were calculated and graphed into time–frequency mapping (Figure 6). The R-means of the mu frequency band and the β frequency band during the movement preparation stage (−1–0 s) and the execution stage (0–2 s) were calculated for statistical analysis. The two-way ANOVA was conducted to test the effect of the movement side (hemiplegic limb movement vs. unaffected limb movement) and locations on the EEG components. The results did not show significant differences in the R-means of either the mu frequency band or the β frequency band among locations, between the movement sides in both rests, or between stages in the CIRM. A paired-sample *t*-test between the R-means of the mu frequency band and the β frequency band showed a statistically significant difference (*T* = −3.304, *p* = 0.001). The mean values of the β-frequency response are higher than those of the mu-frequency response in stroke patients.

## Discussion

In our previous study, 34 healthy participants performed ERP experiments with the same paradigms used in this study. The EEG feature during movement preparation and execution was discussed in our published paper (Li et al., 2018). In this present paper, we focus on the movement-related EEG changes in a stroke patient and refer to some of our previous findings on healthy controls for comparison.

In patients who could perform task movements with paraplegic arms, the EMG peak time was longer in the paraplegic arm than in the unaffected arm, reflecting the impaired movement function. With visual cues, the EMG onset time and the peak time in both arms were shortened, indicating that visual cues could help post-stroke patients with movement preparation and facilitate the initiation of movement. This is consistent with previous studies, which showed that external cues could speed up the motor response in patients with impaired neuromotor function (Praamstra et al., 1998; Amatachaya et al., 2013; Ginis et al., 2018).

In the IRM, the MP amplitude at Cz and the lesional motor cortex were significantly higher during paraplegic arm movement compared to that of the Cz and the contralesional motor cortex during unaffected arm movement. In the CIRM, the same trend was shown but without statistically significant differences, which might be caused by the limited sample size. MP is believed to be the electric excitement that is generated and underlies the motor cortex (Deecke et al., 1969) and that partially reflects the afferent excitement that is generated by the movement (Colebatch, 2007). The MP amplitude may be related to the amount of energy that is required for the movement (Lang et al., 1992). In hemiplegia patients with stroke, on one hand, brain damage obstructed the previously established motor control network connections. To accomplish the same movement task, motor control happens through bypath or reconnection, which reduces the efficiency of the brain motor control and leads to a higher energy demand. On the other hand, efficient neural processing depends on regulating responses through suppression and facilitation of neural activity (Schallmo et al., 2018). It is proposed that generation and inhibition of goal-directed movements emerge from overlapping brain regions in the neural network (Mirabella, 2014; Hampshire and Sharp, 2015), and interactive inhibition occurs from the entire process of movement planning through execution. Thanks to the inhibitory control, the human brain could develop adaptive and flexible behavioral strategies to modulate movements (Mirabella, 2014). During the stoke recovery process, the damaged brain shows not only decreased function of orienting movement but also declined function of movement suppression. The increased MP amplitudes may reflect the combined effects of damage on both facilitation and suppression function. MRCP components were believed to sum the transferable excitatory post-synaptic potentials in apical cortical dendrites (Birbaumer et al., 1990). In healthy subjects, the largest MP amplitude was recorded at Cz (Li et al., 2018). In stroke patients, when the lesional motor cortex consumes more energy and shows higher excitatory potentials during movement, the central area (Cz) summed up the transferable potentials and increased accordingly. The findings on the MP amplitude in stroke patients are consistent with similar studies that were reported in the literature (Wiese et al., 2005; Yilmaz et al., 2013). However, through repeated practice, mastery of certain motor skills results in a lower MP amplitude and longer latency, thereby reflecting better brain motor control efficiency and fewer energy demands (Wright et al., 2011). In stroke rehabilitation, this may explain why better outcomes result from more intensive therapeutic treatment, such as constraint-induced movement therapy (Taub et al., 2006, 2013; Wu et al., 2012; Wolf et al., 2010). With a visual cue, the MP latency was shorter at Cz and the contralesional motor cortex during hemiplegic arm movement in the CIRM than in the IRM. As seen in the EMG data, EMG onset time and peak time were also shortened. This further approved the facilitating effect of a cue before movement initiation. Moreover, the shortened MP latency at Cz and the contralesional motor cortex, but not at the lesional motor cortex, indicates that there is contralateral compensation for brain motor control impairment (Calautti and Baron, 2003; Butefisch et al., 2005). According to stroke motor rehabilitation, it is reasonable to introduce movement cues before initiation of the movement as a common strategy (Shin and Chung, 2017; Ploughman et al., 2018).

In our previous study, in which we investigated brain activity changes during unilateral movements with healthy subjects, the R-means observed mainly in the mu-rhythm band presented bilateral symmetry during movement execution and was the smallest at Cz among the C3, Cz, and C4 locations. In the movement preparation stage, the mu-ERD mainly presented in the sensory–motor cortex area that was contralateral to the movement arm (Figure 6; Li et al., 2018). In the present study, ERD was observed not only in the mu-rhythm band but also in the β-frequency band, with larger mean values. This might have a similar meaning of increased MP amplitude, which indicated more energy consumption during movement planning and execution. The contralateral ERD pattern during movement preparation was not found in the present study in stroke patients. No significant differences were found among investigated locations or between movement sides. Activation of the larger area, including the bilateral motor cortex and the central zone, was observed during both motor preparation and execution stages, with no laterality features. ERD is considered to reflect movement-related brain activity in the time–frequency domain (Neuper et al., 2006), and the findings in the present study indicate that stroke patients produce a larger range of activation in the motor cortex than healthy subjects during movement preparation and execution. This phenomenon also supports the ipsilateral compensation and adjacent-region compensation mechanism in the stroke rehabilitation process that was proposed by previous research using animal models (Starkey et al., 2012; Liu et al., 2019). Although all participants were told to start moving after the GO signal, given the visual cue, a larger motor cortex area was activated in each patient’s brain right after the cue. This phenomenon provides solid evidence for the cued movement therapy strategy for motor rehabilitation. Providing cues may facilitate movement preparation and leads to better and quicker movement performance.

**FIGURE 7:**
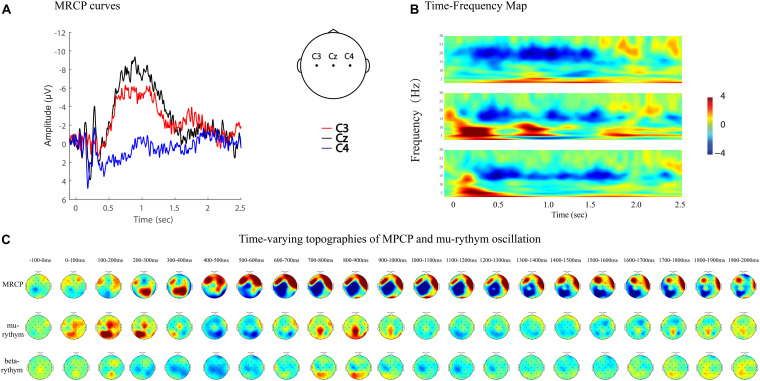
MRCP curves, time-frequency map, and time-varying topographies of MPCP, mu-rythym oscillation of a left hemi-plegic stroke patient during right wrist movements in IRM. **(A)** MRCP curves, negative upwards. **(B)** Time-frequency map. **(C)** Time-varying topographies of MPCP and mu-rythym oscillation, −100 ms to 2000 ms, one topography every 100 ms.

**FIGURE 8:**
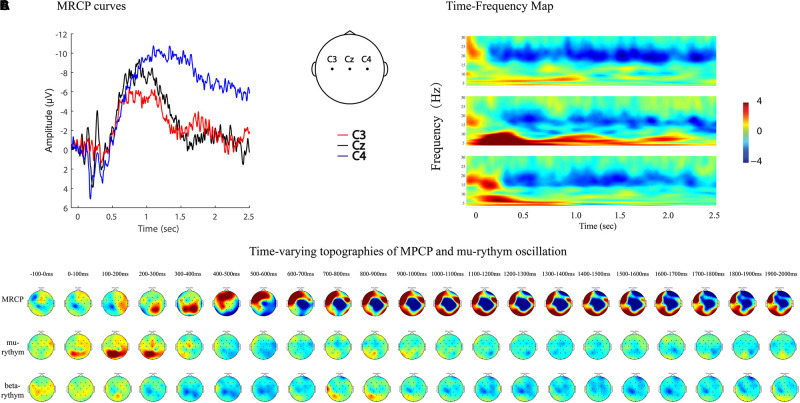
MRCP curves, time-frequency map, and time-varying topographies of MPCP and mu-rythym oscillation of a left hemiplegic stroke patient during left wrist movements in IRM. **(A)** MRCP curves, negative upwards. **(B)** Time-frequency map. **(C)** Time-varying topographies of MPCP and mu-rythym oscillation, −100–2000 ms, one topography every 100 ms.

MRCPs and ERD represent brain activity changes in the time and time–frequency domains. Combining EEG data analyzing techniques in both domains provides more comprehensive information to study brain activity changes in stroke patients. For example, MP amplitude, MP latency, and R-means can be analyzed quantitatively, while regional patterns and time-varying topographies can be used to study the qualitative features of brain activity (Figures 7, 8).

Some EEG-based technology, such as the brain–computer interface (BCI), explored their application in the stroke rehabilitation area. The initial BCI technology studies were based on data from healthy subjects. However, the movement-related brain activity change patterns differ greatly in stroke patients compared to healthy patients, and algorithms that were developed with healthy subjects may not apply to stroke patients. Stroke rehabilitation BCI technology must be based on studies involving stroke patients (Irimia et al., 2017; Mane et al., 2019). The present study provides comprehensive information about the features of movement-related brain activity changes in stroke patients, which may contribute to the research repository. Furthermore, because of the impaired inhibitory control underlining the motor recovery process, researchers should consider how to address the decreased neural suppression and optimize the decision-making process with BCI technology to restore brain motor control functions (Schultze-Kraft et al., 2016; Mirabella and Lebedev Mcapital, 2017).

This study has some limitations. First, the ERD amplitude was reported to correlate with motor impairment and structural damage in stroke patients. However, in this study, we only focused on the EEG data and did not investigate the correlation between EEG features and clinical functions. This might be a meaningful research topic for future study. Second, we only recruited right-handed subjects in this study, and thus, the difference between right-handed and left-handed subjects (Stancak Jr., and Pfurtscheller, 1996) was not investigated. Third, we recruited stroke patients with both cerebral infarction and hemorrhage. Despite the common neurorehabilitation process for stroke patients, there might be different features according to pathological classification, which needs more specific study in the future. Fourth, the patients’ EEG data were analyzed as patients with left or right hemiplegia based on their clinical symptoms and assessment. Because of the variety of clinical manifestations and brain damage, using stroke location as the grouping factor to study the movement-related brain activities will be more precise. We chose the current grouping strategy because of the limited sample size and will consider expanding the sample size and investigating patients’ EEG features according to their stroke location in our future study.

## Conclusion

This study provides comprehensive information on movement-related brain activity changes in stroke patients in both time and time–frequency domains. Quantitative analysis using MP amplitude, MP latency, and R-means, and qualitative analysis of regional patterns and time-varying topographies described the features of post-stroke brain activity changes. The findings support the contralesional compensation and adjacent-region compensation mechanism in the stroke neurorehabilitation process that was proposed by previous animal studies. This study contributes to the research repository and may facilitate future rehabilitation research and technology development, such as the BCI.

## Data Availability Statement

The datasets generated for this study are available on request to the corresponding author.

## Ethics Statement

The studies involving human participants were reviewed and approved by The Ethics Committee at the First Affiliated Hospital of Sun Yat-sen University pre-approved the study protocol before study implementation [(2013)C-068]. The patients/participants provided their written informed consent to participate in this study.

## Author Contributions

HL designed the study and drafted the manuscript. HL and GH analyzed the EEG data and interpreted the results. HL, QL, JZ, LL, and YM conducted the experiments and collected the data. QF and ZZ contributed to EEG data processing and analysis. XW, WY, and BW contributed to data analysis and interpretation. DH supervised all stages of the study including the study design, data collection, analysis, interpretation, and substantial revision of the manuscript. All authors approved the final version of the manuscript and consent for publication.

## Conflict of Interest

The authors declare that the research was conducted in the absence of any commercial or financial relationships that could be construed as a potential conflict of interest.

## Footnotes

1 https://letswave.cn/
