# Supplementary figures and images for: EEG Changes in Time and Time-Frequency Domain During Movement Preparation and Execution in Stroke Patients
Source: Front Neurosci. 2020 Aug 20;14:827. doi: 10.3389/fnins.2020.00827 (PMC7468244; doi:10.3389/fnins.2020.00827)

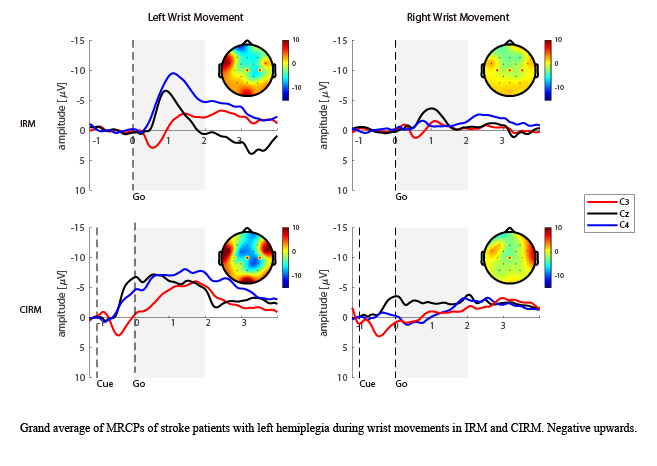

Supplement: Supplementary file 1 [file Image_1.JPEG]

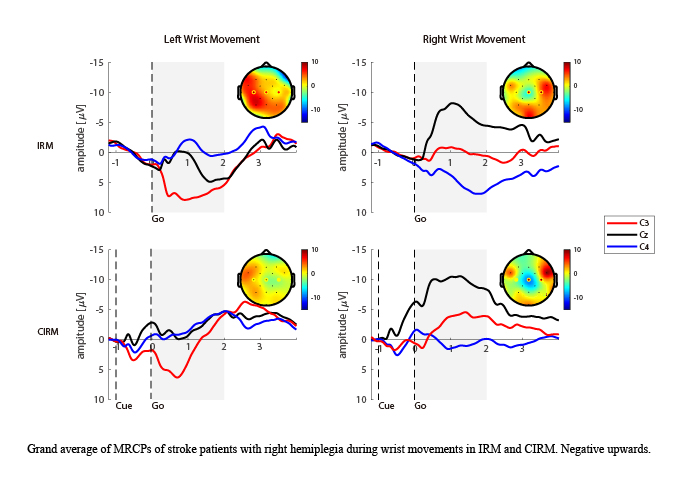

Supplement: Supplementary file 2 [file Image_2.JPEG]
